# Rendfleisch’s Process to Study the Baccilli

**Published:** 1885-02

**Authors:** 


*Rindfleisch's Process to Study the Bacilli.*—Rindfleisch propounds a modification which abbreviates considerably the duration of these manipulations. He slightly warms the coloring liquid. He places for a few seconds above the spirit flame the watch glass containing the solution of fucsine or of violet, until there is noticed a slight vapor on the surface of the liquid; the preparation is then immersed, and in fifteen to twenty minutes the bacilli are sufficiently colored, and the nitric acid solution can be used.

To mount the preparation, Rindfleisch also substitutes for the Canada Balsam a mixture of equal parts of glycerine and a syrupy solution of gum arabic, to which he adds a small quantity of arsenious acid.

The process of Ehrlich, so modified by Rindfleisch, is of sufficiently easy use, and it does not require more than twenty to twenty-five minutes to prepare a specimen. It is also used by Koch, who has renounced his own primitive method. Therefore we can be sure that the bacilli colored with this process are really the bacilli described by Koch, which cannot be affirmed when other methods are employed. The tubercle bacilli are thin, a quarter, half, and may be as much as the diameter of a red globule long, are similar to those of lepra, but thinner and more pointed.

These bacilli are almost always isolated, rarely are united by two at the extremity, and never form masses.

